# Identifying metabolites of new psychoactive substances using in silico prediction tools

**DOI:** 10.1007/s00204-025-04049-5

**Published:** 2025-05-13

**Authors:** Romain Pelletier, Dina Nahle, Mareme Sarr, Alexis Bourdais, Isabelle Morel, Brendan Le Daré, Thomas Gicquel

**Affiliations:** 1 NuMeCan Institute (Nutrition, Metabolisms and Cancer), CHU Rennes, Univ Rennes, INSERM, INRAE, UMR_A 1341, UMR_S 1317, 35000 Rennes, France; 2 Clinical and Forensic Toxicology Laboratory, Rennes University Hospital, 35033 Rennes, France; 3 Pharmacy Department, Rennes University Hospital, 35033 Rennes, France

**Keywords:** New psychoactive substances (NPS), In silico metabolism prediction, Biotransformation pathways, Toxicological biomarkers, Phase I and II metabolism, Prediction software comparison

## Abstract

New psychoactive substances (NPS) pose an increasing challenge for clinical and forensic toxicology due to the initial lack of analytical and metabolic data. This study evaluates the performance of four in silico prediction tools (GLORYx, BioTransformer 3.0, SyGMa, and MetaTrans) in predicting the metabolism of seven NPS from five major chemical families (cathinones, synthetic cannabinoids, synthetic opioids, designer benzodiazepines, and dissociative anesthetics). The predicted metabolites were compared to those reported in the literature. The results revealed that SyGMa was the most exhaustive tool, predicting 437 metabolites, whereas MetaTrans predicted the fewest (61). GLORYx uniquely identified glutathione conjugation, while BioTransformer was particularly effective in predicting phase I reactions. However, no single tool provided complete predictions. Combining the four tools enabled the identification of several key biomarkers consistent with experimental data, such as *m/z* 238.1443 for eutylone and *m/z* 381.1926 for etonitazepipne. These findings highlight the need for integrated approaches to optimize metabolite prediction. Future advancements in artificial intelligence-based models could reduce false positives and enhance the accuracy of predictions, thus reinforcing the role of in silico tools in toxicological investigations.

## Introduction

New psychoactive substances (NPS) comprise a diverse group of recreational drugs (including synthetic cannabinoids, arylcyclohexylamines, synthetic cathinones, new synthetic opiates, and designer benzodiazepines) engineered to replicate the pharmacological effects of illicit drugs such as cannabis, amphetamine, cocaine, 3,4-methylenedioxymethamphetamine, and lysergic acid diethylamide (UNODC 2023). These substances pose a significant challenge in toxicology due to the initial absence of analytical identification data regarding the parent molecule such as exact mass, MS/MS data and retention time. In addition, data on the metabolism of these substances is rarely available, although it is essential as it provides information on the metabolites that serve as consumption markers, particularly in cases where the parent molecule is no longer detected in biological matrices (Gicquel et al. 2024).

The biotransformation reactions of xenobiotics are divided into four main stages: entry of the xenobiotic (phase 0), functionalization reactions (phase I), conjugation reactions (phase II), and exit of the xenobiotic (phase III). In this article, we study the main phase I and II metabolites using NPS metabolism prediction software. NPS metabolism can be explored using a variety of approaches. Among these, human biological samples are considered the gold standard in metabolic studies because they offer real-life insights into the fate of parent compounds and their metabolites. Additionally, analyzing different types of samples provides information on the distribution of compounds and their metabolites in biological matrices. While blood and urine are the most commonly collected samples from living individuals, post-mortem samples can also be collected from bile, gastric contents, cardiac blood, or vitreous humor. The metabolites are known to accumulate particularly in urine and bile, though the limited availability of these samples poses a challenge to broader application (Bardal et al. 2011; Gicquel et al. 2024).

Metabolic studies in animals traditionally rely on rodent models to overcome limitations with human biological samples, but ethical considerations mandate the judicious use of animals (Pelletier et al. 2022a). Despite anatomical, physiological, and biochemical similarities to humans, significant interspecies differences in drug metabolism complicate the extrapolation of rodent, porcine, or canine data to humans (Lin 1995, 1998; Guengerich 1997; Bogaards et al. 2000; Dalgaard 2015).

In vitro studies have corroborated in vivo findings, identifying metabolites also present in human transformation reactions. Liver models, particularly genotyped primary human hepatocytes, are preferred for studying metabolism due to their expression of relevant enzymes and transporters, making them the gold standard despite limitations like high cost and variable enzyme expression (Gerets et al. 2012; Goncalves et al. 2022). Alternative models such as pooled human liver microsomes, pooled human S9 fractions, and differentiated HepaRG cells offer similar results in identifying major metabolites (Gicquel et al. 2024). Overall, several of these models have been employed in NPS metabolism studies using advanced analytical tools to reprocess data, particularly within non-targeted workflow and/or molecular networking (Allard et al. 2019; Pelletier et al. 2022b, 2023, 2024).

To go further, advancements in the understanding of metabolic mechanisms have facilitated the development of in silico metabolism prediction algorithms, which serve as convenient, open-access, time-efficient, and cost-effective tools for expanding metabolite searches and validating in vivo or in vitro data (Kirchmair et al. 2015). Various methodologies are employed in metabolism studies to create in silico systems, including (i) quantitative structure–activity relationship (QSAR) models, which posit that structurally similar molecules exhibit similar metabolic properties, (ii) quantum mechanical calculations for predicting reactivity, and (iii) docking simulations of potential substrates into enzyme active sites (Du et al. 2008; Gertrudes et al. 2012; Kazmi et al. 2019; Tyzack and Kirchmair 2019; Di Trana et al. 2021).

To date, very little data exist on the comparison of in silico xenobiotic metabolism prediction software in general (Boyce et al. 2023), and particularly on NPS. The aim of this study is to compare the results of various in silico metabolism prediction tools on seven NPS candidates belonging to the five most relevant chemical families to compare these tools in the metabolism studies of these new substances.

## Materials and methods

### In silico metabolite prediction

We included seven NPS belonging to the five chemical families most frequently reported in the literature and whose metabolism is accurately described in the literature (Salgueiro-Gonzalez et al. 2024; Santos et al. 2024): cathinones, dissociative anesthetics, synthetic cannabinoids, designer benzodiazepines, and new synthetic opiates (Table 1). The most relevant and up-to-date bibliographic reference was chosen for each NPS.

**Table 1:** NPS selected in this study and classified by chemical family

Family	Name	Canonical SMILES code	Structures
Cathinones	Eutylone	CCC(C(= O)C1 = CC2 = C(C = C1)OCO2)NCC	
	4-Cl-PVP (4-chloro-pyrrolidinovalérophénone)	CCCC(C(= O)C1 = CC = C(C = C1)Cl)N2CCCC2	
Arylcyclohexylamine	2F-DCK (2-fluoro-deschloro-kétamine)	CNC1(CCCCC1 = O)C2 = CC = CC = C2F	
New synthetic opioids (benzimidazoles derivative)	Etonitazepipne (N-Piperidinyl Etonitazene)	CCOC1 = CC = C(C = C1)CC2 = NC3 = C(N2CCN4CCCCC4)C = CC(= C3)[N+](= O)[O−]	
Designer Benzodiazepines	Adinazolam	CN(C)CC1 = NN = C2N1C3 = C(C = C(C = C3)Cl)C(= NC2)C4 = CC = CC = C4	
(Hemi)Synthetic Cannabinoid	HHC (Hexahydrocannabinol)	CCCCCC1 = CC(= C2C3CC(CCC3C(OC2 = C1)(C)C)C)O	
	ADB-Fubinaca	CC(C)(C)C(C(= O)N)NC(= O)C1 = NN(C2 = CC = CC = C21)CC3 = CC = C(C = C3)F	

The NPS metabolites were predicted using their SMILES string obtained in PubChem with four open-access software tools GLORYx, BioTransformer 3.0, SyGMa (Systematic Generation of potential Metabolites) and MetaTrans (Metabolite Translator). All programs predicting the biotransformation of xenobiotics uses reaction rules. They anticipate metabolic pathways and potential metabolites, including phase I and II reactions. However, they differ in their functionalities, which influences the results they deliver.

Among these tools, GLORYx is only available online (https://dev-nerdd.univie.ac.at/gloryx), and allow to export data on.sdf (Structure Data File). GLORYx combines site-of-metabolism prediction via learning models with reaction rules sets to predict and classify potential metabolite structures formed through phase I and/or phase II metabolism (de Bruyn et al. 2021). The “Phase 1 and phase 2 metabolism” parameter was used for GLORYx software.

BioTransformer 3.0 is available online (https://BioTransformer.ca/new) or as a downloadable, command-line only program. It is an open-access tool, enables rapid, accurate, and comprehensive prediction of small molecule metabolism in both mammals and environmental microorganisms (Djoumbou-Feunang et al. 2019). This software combines QSAR models with reaction rules to predict metabolites while identifying the enzymes involved. This feature adds an important biological dimension, facilitating integration with other biological data. For the analysis parameters, we used the ‘AllHuman’ mode, BioTransformer cycle number = 1 and Biotransformer CYP450 prediction Mode = 3.

MetaTrans and SyGMa software packages need to be installed on a computer. In this study, we predicted metabolites with the installed version of software packages using a bash script called Prediction_Metabo.sh (available on https://github.com/alexisbourdais/MetaTox/tree/origin). MetaTrans (Metabolites Translator) uses a deep learning architecture to anticipate metabolic reactions. Its approach relies on precise data, resulting in more targeted predictions. SyGMa (Systematic Generation of potential Metabolites) is a software package that predicts metabolites and biotransformation reactions using a reaction rule-based approach (Ridder and Wagener 2008). It uses predictive models and structural data to estimate the potential metabolites of a chemical compound. SyGMa stands out for its ability to predict a large number of metabolites, covering both phase I and phase II reactions. The parameters used for the SyGMa software are as follows: SyGMa Phase 1 cycle number = 1 and SyGMa Phase 2 cycle number = 1.

### Comparison to literature data

**Fig. 1:**
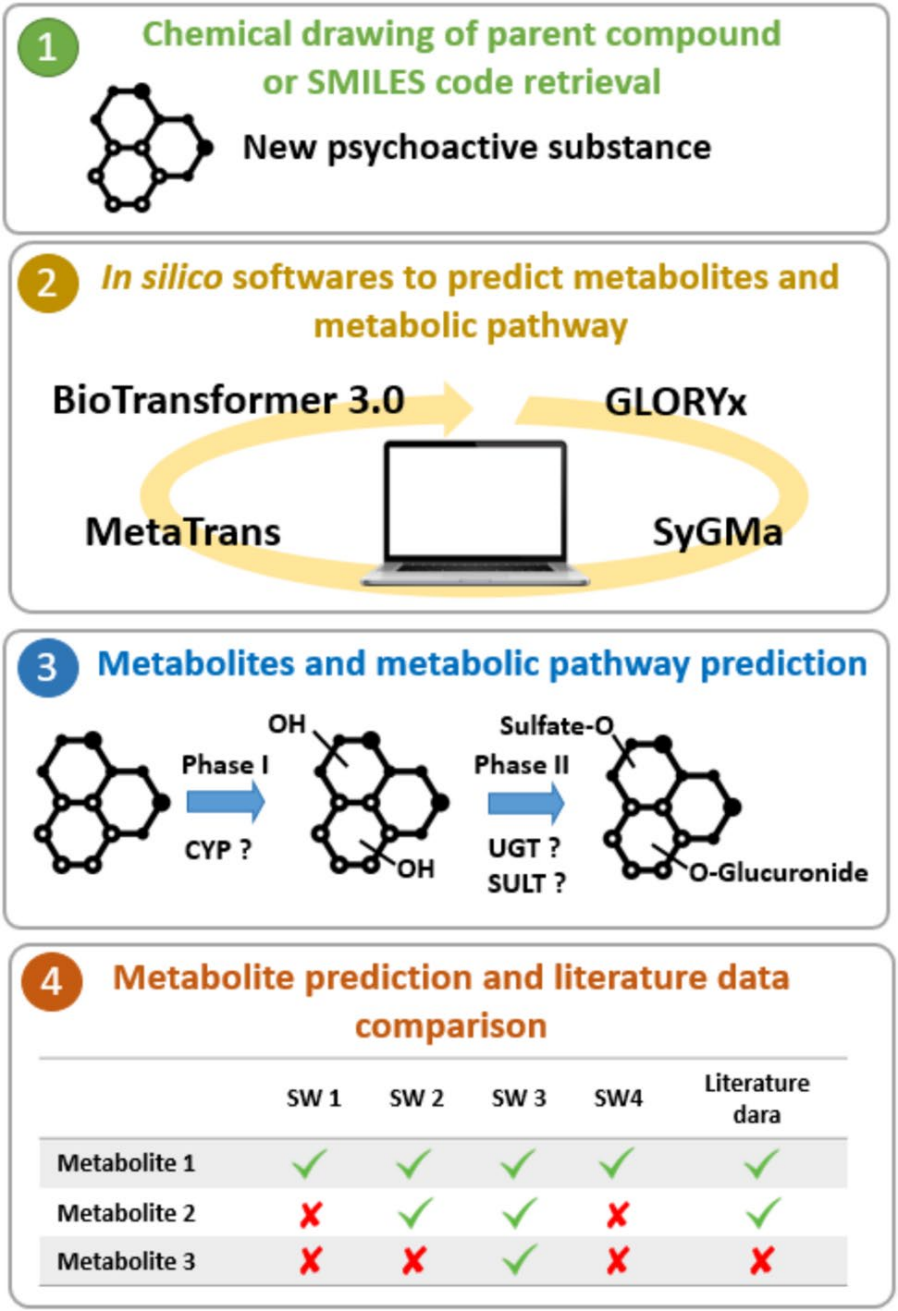
Overview methodology for in silico new psychoactive substances metabolism prediction used in this study. OH: hydroxylation; CYP: cytochrome P450; SULT: sulfotransferase; SW: software; UGT: UDP-glucuronosyltransferase

A literature review was carried out on the PubMed, Google Scholar, and Web of Science databases to select studies presenting data on NPS metabolism. The following molecules were identified and selected for this study on the basis of their known metabolism in the literature: 2 synthetic cathinones (eutylone and 4-Cl-PVP), 1 dissociative anesthetic (2F-DCK), 1 synthetic cannabinoid (ADB-Fubinaca), 1 semi-synthetic cannabinoid (HHC), 1 designer benzodiazepine (adinazolam) and 1 new synthetic opiate (etonitazepipne). An overview of the methodology used in this study is shown in Fig. 1.

The lists of NPS metabolites presented in Tables 4, 5, 6, 7, 8, 9 and 10 were compiled by integrating metabolites from two sources: experimentally confirmed metabolites reported in the literature, and theoretical metabolites predicted by in silico tools. The tables below, therefore, present only those molecules found by the software and already described in the literature from relevant references that have studied the metabolism of the molecule of interest, using human samples and/or, where appropriate, in vitro models.

### Chemical structures drawing

All the molecules were drawn using Chemdraw 23.1.1 software. To standardize the structures, we used the ACS mode of document 1996.

## Results

### In silico metabolism prediction overview

The metabolism of the seven NPS selected in this study was predicted using four in silico prediction software packages. Table 2 summarizes the total number of metabolites predicted by each software package, as well as the distribution between phase I and phase II metabolites. The results varied significantly across the different software tools. SyGMa predicted the highest number of metabolites (437), followed by GLORYx (191), BioTransformer (91), and MetaTrans (80).

**Table 2:** Summary of predicted metabolites for each compound based on in silico metabolism prediction software

Parent compound	Number of predicted metabolites Total (phase I + Phase II)			
	GX	BT	MT	SM
Eutylone	34 (34 + 0)	11 (11 + 0)	9 (9 + 0)	46 (23 + 23)
4-Cl-PVP	27 (26 + 1)	15 (15 + 0)	14 (14 + 0)	28 (11 + 17)
2-FDCK	5 (5 + 0)	10 (10 + 0)	10 (10 + 0)	40 (26 + 14)
Etonitazepipne	38 (37 + 1)	18 (18 + 0)	12 (12 + 0)	132 (50 + 82)
Adinazolam	27 (24 + 3)	12 (11 + 1)	9 (9 + 0)	69 (22 + 47)
HHC	34 (31 + 3)	16 (14 + 2)	10 (10 + 0)	72 (29 + 43)
ADB-Fubinaca	26 (26 + 0)	9 (8 + 1)	16 (16 + 0)	50 (16 + 34)
Total	191 (183 + 8)	91 (87 + 4)	80 (80 + 0)	437 (177 + 260)

GX: GLORYx; BT: BioTransformer; SM: SyGMa; MT: MetaTrans

Among the four software packages, MetaTrans predicted the fewest metabolites and did not predict any phase II metabolites. BioTransformer 3.0 predicted between 9 and 18 metabolites per NPS but identified phase II metabolites for only three of the seven molecules, resulting in a total of 91 metabolites. GLORYx predicted between 5 and 38 metabolites per NPS but included only eight phase II metabolites out of a total of 191 metabolites. SyGMa provided the broadest range of predicted metabolites, particularly due to its higher number of phase II metabolites. Specifically, SyGMa predicted more phase II metabolites (260) than phase I metabolites (177) when aggregating the results for all molecules.

In terms of biotransformation reactions, phase I reactions such as hydroxylation/oxidation and dealkylation were predicted by all the software packages. However, BioTransformer was the only software that did not predict carboxylation. All software packages, except MetaTrans, were capable of predicting phase II metabolites (Table 3). Sulfation reactions were predicted exclusively by SyGMa and GLORYx. Additionally, glutathione conjugates were predicted only by GLORYx for 4-Cl-PVP and Adinazolam. Overall, these results indicate that MetaTrans provides the least diversity in terms of biotransformation reactions.

**Table 3:** Biotransformation metabolism pathway predicted by each software package

Molecule	BioTransformer	MetaTrans	Sygma	GLORYx
**Eutylone**	**Phase I** Reduction Hydroxylation Oxidation Dealkylation	**Phase I** Dealkylation Hydroxylation Oxidation	**Phase I** Dealkylation Reduction Hydroxylation Carboxylation **Phase II** Sulfation Glucuronidation	**Phase I** Dealkylation Oxidation Hydroxylation Carboxylation Reduction
**4-Cl-PVP**	**Phase I** Reduction Hydroxylation Oxidation Dealkylation Desaturation/reduction	**Phase I** Hydroxylation Dealkylation Carboxylation	**Phase I** Reduction Hydroxylation Oxidation Carboxylation **Phase II** Glucuronidation Sulfation	**Phase I** Hydroxylation Reduction Dealkylation Oxidation Dehydrogenation/reduction Carboxylation Dechlorination **Phase II** Glutathione conjugation
**2F-DCK**	**Phase I** Hydroxylation Dealkylation Reduction	**Phase I** Hydroxylation Dealkylation Reduction	**Phase I** Demethylation Reduction Hydroxylation **Phase II** Glucuronidation Sulfation Acetylation	**Phase I** Hydroxylation Dealkylation
**Etonitazepipne**	**Phase I** Reduction (nitroreduction) Hydroxylation Dealkylation Oxidation Iminium formation Cycle opening	**Phase I** Hydroxylation Oxidation	**Phase I** Hydroxylation Dealkylation Reduction **Phase II** Glucuronidation Sulfation	**Phase I** Hydroxylation Dealkylation Oxidation Dehydration/ reduction Dehydrogenation/reduction **Phase II** Glucuronidation
**Adinazolam**	**Phase I** Hydroxylation Oxidation Dealkylation **Phase II** Glucuronidation	**Phase I** Demethylation Dealkylation Hydroxylation Decarbonation	**Phase I** Demethylation Oxidation Hydroxylation Dealkylation Dechlorination Imine-Hydrolyse **Phase II** Glucuronidation Sulfation	**Phase I** Hydroxylation Demethylation Oxidation Dealkylation Dehydration Dechlorination Imine-Hydrolyse **Phase II** Glucuronidation Glutathione conjugation
**HHC**	**Phase I** Hydroxylation Desaturation/reduction **Phase II** Glucuronidation Sulfation Methylation	**Phase I** Oxidation Demethylation	**Phase I** Hydroxylation **Phase II** Glucuronidation Sulfation Methylation Carboxylation	**Phase I** Hydroxylation Dehydrogenation Oxidation **Phase II** Glucuronidation Sulfation Carboxylation Methylation
**ADB-Fubinaca**	**Phase I** Hydroxylation **Phase II** Glucuronidation	**Phase I** Hydroxylation Dealkylation	**Phase I** Hydrolysis Hydroxylation Dealkylation **Phase II** Glucuronidation Sulfation	**Phase I** Dealkylation Oxidation Hydroxylation Hydrolysis **Phase II** Glucuronidation

### Metabolism prediction compared with literature data

#### Eutylone

Pelletier et al. (2023) identified 16 metabolites of eutylone using samples from two human patients (blood and urine) who had consumed eutylone. Among these, the authors described a phase I metabolite (*m/z* 238.144) and 2 glucuronoconjugated metabolites (*m/z* 400.160 and *m/z* 414.176) as reliable markers of eutylone consumption.

In comparison, the four in silico prediction software packages used in this study predicted 7 of the 16 metabolites identified by the reference study. Decarbonated metabolite (*m/z* 224.1286) and reduced metabolite (*m/z* 238.1443) were consistently identified by all four software packages. Notably, SyGMa was the only software capable of predicting the metabolite with *m/z* 250.1079, as well as phase II metabolites, including those identified as markers of eutylone consumption (*m/z* 400.1607 and *m/z* 414.1764) (Tables 4, 5, 6, 7, 8, 9 and 10).

**Table 4:** Eutylone metabolite prediction using in silico software compared with literature data (Pelletier et al. 2023) (– = not applicable; X = present; Gluc = glucuronic acid; Sulf = sulfate)

Molecule Formula [M + H]^+^(*m/z*)	Mass shift	Chemical structure Biotransformation	GX	BT	MT	SM	In vivo (human blood)
Eutylone C_13_H_17_NO_3_ 236.1287	–		**–**	**–**	**–**	**–**	**–**
C_12_H_17_NO_3_ 224.1286	− 12.0000		X	X	X	X	X
C_13_H_19_NO_3_ 238.1443	+ 2.0156		X	X	X	X	X
C_13_H_19_NO_3_ 238.1443	+ 2.0156		X	X	X	X	X
C_13_H_15_NO_4_ 250.1079	+ 13.9792					X	X
C_12_H_17_NO_6_S 304.0854	+ 67.9567					X	X
C_13_H_19_NO_6_S 318.1011	+ 81.9725					X	X
C_18_H_25_NO_9_ 400.1607	+ 164.032					X	X
C_19_H_27_NO_9_ 414.1764	+ 178.0477					X	X

**Table 5:** 4-Cl-PVP metabolite prediction using in silico software compared with literature data from in vivo (human blood and urine) and in vitro studies (HepaRG cell line) (Pelletier et al. 2022b) (− = not applicable; X = present; gluc = glucuronic acid)

Molecule Formula [M + H]^+^ (*m/z*)	Mass shift	Chemical structure Biotransformation	GX	BT	MT	SM	In vitro (HepaRG cells)	In vivo (human blood and urine)
4-Cl-PVP C_15_H_20_ClNO 266.1312	NA		**–**	**–**	**–**	**–**	**–**	**–**
M4 C_15_H_18_ClNO_2_ 280.1104	+ 13.9792		X			X	X	X
M5 C_15_H_20_ClNO_2_ 282.1260	+ 15.9948		X	X	X	X	X	X
M7 C_15_H_18_ClNO_3_ 296.1053	+ 29.9741		X			X	X	X
M8 C_15_H_20_ClNO_3_ 298.1209	+ 31.9897					X	X	X
M5-Gluc C_21_H_28_ClNO_8_ 458.1581	+ 192.0269					X		X
M7-Gluc C_21_H_26_ClNO_9_ 472.1374	+ 206.0062					X		X

**Table 6:** 2F-DCK metabolite prediction using in silico software compared with literature data from in vitro (Human Liver Microsomes (HLMs), HepaRG cell line) and post-mortem samples (Gicquel et al. 2021) (– = not applicable; X = present; gluc = glucuronic acid)

Molecule Formula [M + H]^+^ *(m/z*)	Mass shift	Structure Biotransformation	GX	BT	MT	SM	In vitro (HLMs/HepaRG cells)	In vivo (*post-mortem* samples)
2F-DCK C_13_H_16_FNO 222.1286	NA		–	–	–	–	–	–
Hydroxy-2F-DCK M01 or M03 or M19 C_13_H_16_FNO_2_ 238.1238	+ 15.9952		X	X	X	X	X/X	X
Nor-2F-DCK M09 C_12_H_14_FNO 208.1130	− 14.0156		X	X	X	X	X/X	X
Dihydro-2F-DCK M12 or M15 C_13_H_18_FNO 224.1442	+ 2.0156			X	X	X	X/X	X
Hydroxy-nor-2F-DCK glucuronide M18 C_18_H_22_FNO_8_ 400.1398	+ 178.0112					X		X
Hydroxy-2F-DCK glucuronide M20 C_19_H_24_FNO_8_ 414.1558	+ 192.0272					X		X

**Table 7:** Etonitazepipne metabolite prediction using in silico software compared with literature data from post-mortem urine samples (Berardinelli et al. 2024a) (−  = not applicable; X = present; gluc = glucuronic acid). HLMs = human liver microsomes

Molecule Formula [M + H]^+^ (*m/z*)	Mass shift	Structure Biotransformation	GX	BT	MT	SM	In vitro (HLM)	In vivo (*post-mortem* urines)
Etonitazepipne C_23_H_28_N_4_O_3_ 409.2239	–		–	–	–	–	–	–
M1 C_23_H_30_N_4_O 379.2497	− 29.9742		X		X	X	X	X
M3 C_27_H_32_N_4_O_9_ 557.2247	+ 148.0008					X		X
M4 or M8 C_21_H_24_N_4_O_4_ 397.1876	− 12.0363		X				X	X
M10 C_21_H_24_N_4_O_3_ 381.1926	− 28.0313		X	X	X	X	X	X
M11 or M21 C_23_H_28_N_4_O_4_ 425.2188	+ 15.9949		X	X	X	X	X	X
M13 or M16 or M18 C_23_H_28_N_4_O_4_ 425.2188	+ 15.9949		X	X	X	X	X	X
M14 C_29_H_36_N_4_O_10_ 601.2509	+ 192.0270					X		X
M15 C_24_H_27_N_3_O_10_ 518.1774	+ 108.9535					X		X
M17 C_23_H_28_N_4_O_5_ 441.2138	+ 31.9899			X			X	X
M23 C_23_H_26_N_4_O_4_ 423.2032	+ 13.9793		X			X	X	X

**Table 8:** Adinazolam metabolite prediction using in silico software compared with literature data from patient urine (Fraser et al. 1993) (– = not applicable; X = present)

Molecule Formula [M + H]^+^ (*m/z*)	Mass shift	Structure Biotransformation	GX	BT	MT	SM	In vivo (human urines)
Adinazolam C_19_H_18_ClN_5_ 352.1329	NA		-	-	-	-	-
N-desmethyladinazolam C_18_H_16_ClN_5_ 338.1172	− 14.0157		X	X	X	X	X
OH-alprazolam C_17_H_13_ClN_4_O 325.0856	− 27.0473		X		X	X	X

**Table 9:** Hexahydrocannabinol metabolite prediction using in silico software compared with literature data from patient urine (Lindbom et al. 2024) (– = not applicable; X = present; gluc = glucuronic acid)

Molecule Formula [M + H]^+^ (*m/z*)	Mass shift	Structure Biotransformation	GX	BT	MT	SM	In vitro (human hepatocytes)	In vivo (human urines)
HHC C_21_H_33_O_2_ 317.2481	–		–	–	–	–	–	–
N1 C_27_H_40_O_8_ 493.2801	+ 176.0320		X	X		X		X
N2 C_27_H_40_O_9_ 509.2750	+ 192.0269					X	X	X
N3 C_27_H_40_O_9_ 509.2750	+ 192.0269					X	X	X
N6 C_27_H_38_O_10_ 523.2543	+ 206.0062					X	X	X
N7 C_21_H_30_O_4_ 347.2222	+ 29.9741		X		X	X	X	X
N10 C_21_H_32_O_3_ 333.2429	+ 15.9948		X	X	X	X		X

**Table 10:** ADB-Fubinaca metabolite prediction using in silico software compared with literature data from pooled human hepatocytes (Carlier et al. 2017) (– = not applicable; X = present; gluc = glucuronic acid)

Molecule Formula [M + H]^+^ (*m/z*)	Mass shift	Structure Biotransformation	GX	BT	MT	SM	In vitro (pooled human hepatocytes)
ADB-Fubinaca Parent C_21_H_23_FN_4_O_2_ 383.1883	NA		-	-	-	-	-
M14 C_21_H_23_N_4_O_3_F 399.1832	+ 15.9949		X	X	X	X	X
M16 C_21_H_23_N_4_O_3_F 399.1832	+ 15.9949		X	X	X	X	X
M3 C_14_H_18_N_4_O_2_ 275.1509	− 108.0374		X		X	X	X
M11 C_21_H_23_N_4_O_4_F 415.1791	+ 31.9898				X		X
M10 C_27_H_31_N_4_O_9_F 575.2156	+ 192.0263					X	X
M18 C_27_H_30_N_3_O_9_F 560.2044	+ 177.0161		X			X	X
M22 C_21_H_22_N_3_O_3_F 384.1726	+ 0.9843		X			X	X

#### 4-Cl-PVP

Pelletier et al. (2024) identified 15 metabolites of 4-Cl-PVP using human biological samples (blood and urine) from a case of 4-Cl-PVP poisoning, as well as in vitro studies on HepaRG cell line. Among these metabolites, M5 (a hydroxy derivative) and M8 (a dihydroxy derivative) were highlighted as reliable markers of 4-Cl-PVP consumption.

In our study, only 6 of the 15 metabolites described in the reference study were predicted by combining the four in silico prediction software packages. Metabolite M5 was identified by all four software tools, consistent with its prominence in the reference study. However, BioTransformer and MetaTrans each predicted only one metabolite in common with the reference study (M5). GLORYx predicted three already described metabolites: M4, M5, and M7. Notably, SyGMa was the only software capable of predicting M8 and several other metabolites described in the reference study.

#### 2F-DCK

Gicquel et al. (2024) identified 20 metabolites of 2F-DCK using *post-mortem* human samples (blood, vitreous humor, bile, and urine) from a case of 2F-DCK poisoning, as well as in vitro studies on human liver microsomes (HLMs) and HepaRG cell line. Among these metabolites, nor-2F-DCK (M09, *m/z* 208.1130) and dihydro-2F-DCK (M12, *m/z* 224.1443) were proposed as reliable metabolites to be recorded in HRMS libraries to improve detection of 2F-DCK.

In our study, the four in silico prediction software packages were able to predict eight metabolites. These included six phase I metabolites (M01, M03, M19, M09, M12, and M15) and two phase II metabolites (M18 and M20). Notably, only the SyGMa software was able to predict phase II metabolites. Metabolite M09 was predicted by all four software packages, whereas metabolite M12 was predicted by three of them.

#### Etonitazepipne

Berardinelli et al. (2024a, b) identified 23 metabolites of etonitazepipne using post-mortem urine samples from a case of etonitazepipne poisoning, as well as in vitro studies on HLMs. In the urine sample, the most abundant metabolites were M10 (O-dealkylation) and M20 (O-dealkylation and oxidation), while in the HLMs, the most abundant metabolites were M10 (O-dealkylation) and M5 (O-dealkylation and oxidation).

Of the 23 metabolites described by Berardinelli et al. (2024a, b), the four software packages were able to predict 14 metabolites, including phase I metabolites M10, M11, M13, M14, M16, M17, and M18 (demethylated and hydroxylated derivatives). Metabolite M17 was predicted only by BioTransformer, corresponding to a carboxylation reaction. Phase II metabolites were predicted exclusively by SyGMa, corresponding to the glucuronoconjugate derivatives M14 and M15. Of the most abundant metabolites described in the reference publication, metabolite M09 was predicted by all four software packages. Of the other metabolites predicted by the four software packages (M11, M13, M16, M18, and M21), only M13, M18, and M21 were detected in the urine of the intoxicated patient.

#### Adinazolam

Fraser et al. (1993) identified four adinazolam metabolites (N-desmethyladinazolam, α-OH-alprazolam, estazolam and *N,N*-didesmethyladinazolam) in the urine of six volunteers given single oral doses of 10, 30, and 50 mg adinazolam. Urine samples were collected from 0 to 36 h post-administration (Fraser et al. 1993).

Among these metabolites, the four software packages were able to predict two common metabolites. There were two phase I metabolites, including N-desmethyladinazolam predicted by GLORYx, BioTransformer, SyGMa and MetaTrans, and hydroxyl-adinazolam predicted by GLORYx SyGMa and MetaTrans. No phase II metabolites were found in the publication by Fraser et al. (1993) unlike in silico predictions (Table 3).

#### Hexahydrocannabinol

Lindbom et al. (2024) identified 21 HHC metabolites in 16 authentic patient urine samples. HHC was primarily metabolized through monohydroxylation, followed by oxidation to a carboxylic acid metabolite.

Of these 21 metabolites, 11 were predicted in silico, eight of which were phase II metabolites. Most of these metabolites were only predicted by SyGMa and only N10 metabolite was predicted by all four software packages simultaneously. N1 corresponding to glucuronoconjugation of HHC, and N7 corresponding to hydroxylation were found by three software packages, simultaneously.

#### ADB-Fubinaca

Carlier et al. (2017) identified 23 metabolites of ADB-Fubinaca after 1 and 3 h of incubation with pooled human hepatocytes. The main metabolic pathways included alkyl and indazole hydroxylation, terminal amide hydrolysis, subsequent glucuronide conjugations, and dehydrogenation. Consequently, ADB–Fubinaca hydroxyalkyl (M16), hydroxydehydroalkyl (M15), and hydroxylindazole (M14) metabolites were proposed as potential markers for ADB-Fubinaca intake.

Of these 23 ADB-Fubinaca metabolites, 7 were predicted in silico. Only 2 metabolites were predicted in the same way by all software packages, namely M14 and M16. Metabolite M3 (dealkylated) was also predicted by three software packages (all except BioTransformer).

## Discussion

In this study, we aimed to compare the results of four in silico metabolism prediction software packages on 7 NPS candidates belonging to five different chemical families to better position these tools in the metabolism studies of these new substances. Indeed, in silico prediction software are useful tools to explore the metabolism of NPS (Pelletier et al. 2022a, 2024). By combining biotransformation reaction modeling with databases and learning algorithms, these software packages offer innovative perspectives for describing potential metabolites, thus reducing the time and costs associated with in vitro and in vivo studies. Also these software packages are freely available and easy to use, despite their performance varies considerably depending on the software used.

GLORYx predicted a total of 191 metabolites, covering both phases I and II biotransformations. Interestingly, GLORYx proposed less common predictions, such as glutathione conjugation for Adinazolam and 4-Cl-PVP, demonstrating its potential for exploring complex metabolic pathways. Lastly, while GLORYx proposes ranks and scores to prioritize metabolic scenarios and support metabolite identification performed in vitro or in vivo as already used in the literature (Di Trana et al. 2021; Berardinelli et al. 2022, 2024b; Pelletier et al. 2022b; Brunetti et al. 2023), we included in this study all the proposed metabolites to be able to compare the software packages with each other.

With 91 predicted metabolites, BioTransformer 3.0 positions itself as a balanced tool, predicting major reactions such as hydroxylation and dealkylation for most of the evaluated NPS. In particular, this advantage has been used in the literature to add the expected main metabolites to the inclusion lists during analysis by high-performance liquid chromatography–high-resolution mass spectrometry (Verougstraete et al. 2023). However, BioTransformer sometimes lacks precision in certain predictions. For instance, it failed to identify glucuroconjugated metabolites for eutylone and 2F-DCK, even though these are often essential biomarkers in toxicological studies (Gicquel et al. 2021; Pelletier et al. 2023).

MetaTrans predicted the fewest number of metabolites (*n* = 80), without including any phase II reactions for the evaluated substances. This limitation reduces its relevance for comprehensive exploration of NPS metabolism. Nevertheless, MetaTrans proved its value by predicting certain key metabolites, such as Nor-2F-DCK for 2F-DCK, a relevant biotransformation identified in the literature (Gicquel et al. 2021). This demonstrates that even with a limited number of results, the quality of the predictions remains significant.

With 437 predicted metabolites for the seven NPS studied, SyGMa offers the broadest range, particularly for conjugation reactions such as glucuronidation and sulfation. However, this advantage can also be a drawback when the software predicts redundant or aberrant metabolites. In the 2F-DCK example, SyGMa proposed 10 metabolites but only three unique biotransformations, complicating data analysis. Its exhaustiveness can thus result in additional work to filter relevant results.

In terms of overall coverage, SyGMa emerges as the most exhaustive tool, although this can result in superfluous predictions. MetaTrans and BioTransformer focus more on precise but limited predictions, which can be advantageous for targeted studies. GLORYx, with its balance between coverage and relevance, offers an interesting solution based on these results.

Our findings indicate that SyGMa is particularly effective in generating phase II metabolites for all tested molecules. However, combining multiple tools significantly enhanced the metabolite coverage. To predict reliable biomarkers in silico, the most robust approach involves integrating several software tools, as demonstrated in previous studies (Pelletier et al. 2022a, b, 2024). This integrative approach minimizes aberrant metabolites and streamlines the selection of relevant candidates from extensive predictions. By addressing individual inconsistencies, the combined use of the four software tools identifies metabolites with the highest likelihood of occurring in vivo when consistently predicted across all tools (Gicquel et al. 2024).

As part of this study’s limitations, it is likely that certain metabolites identified in silico are indeed present in biological samples but were not detected in the studies. It would, therefore, be appropriate to carry out other in vitro or in vivo analyses using the in silico data generated here. Conversely, we show here that in silico software cannot always suggest metabolites of interest. In the example of adinazolam, the software predicts between 9 and 69 metabolites. In the literature (Fraser et al. 1993), four metabolites were identified as being of biomarker interest, but only two of these were suggested by the software, leaving 50% of the relevant metabolites unidentified. *N,N*-didesmethyladinazolam is not proposed, although it seems relevant that it should be found, given the chemical structure of this benzodiazepine. Among other drug classes, *N,N*-didesmethyl-derivatives are notably found with tramadol and venlafaxine.

With regard to synthetic cathinones (eutylone and 4-Cl-PVP), some metabolites identified as potential consumption markers in the literature were predicted by the four software packages, such as *m/z* 238.1443 for eutylone and M5 for 4-Cl-PVP. These results might suggest that the prediction of metabolites of other cathinones by these four software packages could lead to the proposal of relevant consumption markers to look for even before obtaining in vivo or in vitro samples. Similar results were obtained with three other molecules for which the metabolites predicted in common by the four software packages constitute proposed n markers, such as M09 for 2F-DCK, M10 for etonitazepipne and M14/M16 for ADB-Fubinaca, in vitro or in vivo consumption.

Overall, although this work enables us to evaluate the performance of four metabolic prediction software packages, we show that these tools cannot yet replace in vitro and in vivo experiments to identify relevant biomarkers. Looking ahead, the development of next-generation metabolic prediction tools with lower false-positive rates could further refine predictions, enhancing the identification of biomarkers in the absence of in vitro or in vivo studies. Promising examples include Metapredictor (Zhu et al. 2024) and Semeta (https://optibrium.com/products/semeta/) whose metabolite prediction should be studied in the light of the literature. Future advancements in machine learning and the integration of artificial intelligence are thus expected to yield more accurate and specific predictions.

## Conclusion

In silico prediction tools offer a valuable, cost-effective, and efficient approach to explore the metabolism of new psychoactive substances (NPS), particularly in the absence of biological samples. SyGMa demonstrated the most comprehensive coverage, especially in predicting phase II metabolites, while BioTransformer 3.0 and MetaTrans provided more targeted but narrower predictions. GLORYx stood out for its innovative pathways, such as glutathione conjugation, although these conjugates have not been identified in the literature. Combining multiple tools proved critical to overcoming individual limitations and enhancing prediction reliability, as demonstrated by the consistent identification of key metabolites such as *m/z* 238.1443 for eutylone and *m/z* 381.1926 for etonitazepipne. This integrative approach increases the likelihood of identifying reliable biomarkers of consumption. Looking forward, the integration of artificial intelligence and machine learning into next-generation tools promises to improve accuracy, reduce false positives, and better predict complex metabolic pathways. By bridging the gaps in current methods, in silico tools are poised to play a pivotal role in toxicological investigations, supporting early detection and characterization of emerging NPS.
